# Shall Nurses Cease to Be Laywomen?—V

**Published:** 1891-10-10

**Authors:** 


### PASSING TOPICS.

### SHALL NURSES CEASE TO BE LAYWOMEN?—V.

*(Concluded.)*

When we are told that no less than fourteen hundred applications for probationerships in excess of requirements were received in one year by a single hospital, we can appreciate the independence of the institutions and understand how unnecessary it is to hold out inducements beyond those which are legitimate. There is no need to offer meretricious attractions of any sort, and above all things young women should not be induced to enter the ranks by romantic pictures of a nurse's life, which are as pertinent to the real round of duty as the ribbons of the recruiting sergeant to the goose step and platoon drill. There is no romance, but much hard and painful work such as ought to be approached in a grave and womanly spirit, and with some consciousness of the sanctity of the occupation about to be entered upon.

We take it that the nursing of the sick is, and must be, more or less a self-sacrificing pursuit, and that the best teacher and chief for aspirants is not a physician or surgeon, but a competent, earnest, and let us add, devout woman. Probably the very highest type of nurse is found among the members of those religious bodies where the individual is merged in the Sisterhood, where sometimes money, sometimes rank, and always the good things of this world, are surrendered, and even her name is obliterated. We do not say that effacement like this is necessary, any more than we suppose that women in sufficient numbers would be willing to accept it. But self-consciousness is a terrible, almost an unpardonable, fault in a nurse, and if she is to succeed she must be capable of forgetting herself. She must live in and for her work. Moreover, she must regard it as a work of compassion. To the doctor, the patients are cases; they ought to be more than that to the nurse. The nurses must be willing subordinates, obedient in the minutest particular to medical orders, but capable of superadding something of their own. An ideal nurse is not content with avoidance of neglect. She is the good genius of the ward; her words raise the drooping heart; she is always in touch with her patients. Neither is she altogether forgetful of an hereafter, nor content to accept and repeat the commonplaces of scientific scepticism. Religion is more to her than a superstition, and the visit of the chaplain is as welcome as that of the doctor himself.

Whoever wrote the first Sunday Fund Number of The Hospital revealed a glimpse of the beautiful when he gave us the evening picture of the Sister sweetly singing "Rock of Ages," in the hospital ward. Let us be thankful to know the scene was no mere effort of imagination, and that in many a ward we have as the central figure a devoted woman, who is indeed the "ministering angel" we delight to think her. We do not say that this presence would be impossible in an atmosphere wholly scientific, but it would exist if at all as a survival and an anachronism—always liable to fall under the the ban of the professional martinet.

Does it never occur to those who are satisfied to look upon the nurse's occupation as a species of dextrous drill and sleight of hand, and upon the patients as types and cases, what it is to suffer, perhaps to die—where at the best, routine and duty are the substitutes for love and death itself is regarded with a purely professional and abstract view? Nurses, ambitious of a professional standing, should ponder on these things, and they would come to see what it is they are proposing to barter for a mock diploma. It is alleged of women that they never attain to originality; that in their greatest eccentricities of dress, of occupation, or of pastime they aspire only to imitate. Let our nurses at least prove the exception, and in their service to the sick let them be prodigal of their womanhood.

The absorption of nurses into the medical following would mean the loss of most of the amiabilities, and it may be safely predicted that, under such conditions, hospitals, as we know them, would cease to exist, and the grand old title of Maison de Dieu, so eloquent in its graphic simplicity, would be expunged for ever. Some other method of maintenance than by benefactions would have to be devised, while the patients would grow chary of committing themselves to the care of institutions devoted to the pursuit of science alone. The question of a nursing profession hedged about by technicalities too dense for penetration by the sweetening influences of philanthropy is very far from one which concerns the nurses only.

Agricola.

